# The Interactions of Absorptive Capacity, Buffer Inventory, and Toxic Emissions on Firm Value

**DOI:** 10.3390/ijerph18041979

**Published:** 2021-02-18

**Authors:** Lik Man Daphne Yiu, Ka Yui Karl Wu

**Affiliations:** 1 School of Business, Macau University of Science and Technology, Macau S.A.R, China; lmyiu@must.edu.mo; 2 School of Business, Singapore University of Social Sciences, Singapore 599494, Singapore

**Keywords:** absorptive capacity, buffer inventory, toxic emissions, firm value

## Abstract

A significant amount of research has been conducted on the impacts of emissions reduction, absorptive capacity, and buffer inventory on firm performance. According to the resource-based view (RBV), absorptive capacity and buffer inventory are organizational capabilities and resources to create sustainable competitive advantages. Yet, the resource orchestration perspective (ROP) of the RBV emphasizes that firms need to develop a new capability to orchestrate and deploy their existing capabilities and resources. From an organizational learning perspective, firms with the low-level release of toxic chemicals have established a structured system and systematic organizational routines, strengthening their learning capabilities to share and use internal and external information across functional areas for continuous improvements. This study explores and seeks to understand toxic emissions through systematic operational routines as an organizational mechanism. These routines orchestrate and deploy the firm-specific absorptive capacity and buffer inventory to generate a sustainable competitive advantage. We examine the impacts of the absorptive capacity and buffer inventory on firm value in terms of Tobin’s Q, respectively. We also explore how such impacts are moderated by toxic emissions. Our results show that the absorptive capacity significantly enhances the market value of firms. However, the relationship between the buffer inventory and firm value is insignificant. Our additional analyses indicate that the impacts of the absorptive capacity and buffer inventory on the firm value are both significantly positive when firms release low toxic chemicals. Our results further suggest that firms can maximize their market value with a high absorptive capacity, high buffer inventory, and low toxic emissions.

## 1. Introduction

Faced with the complex and unpredictable changing environment, firms are interested in developing environmental practices to respond to growing regulatory requirements, variations in customer demands, compliance costs, and risks to a corporate’s reputation [1,2]. Manufacturing processes could produce a huge amount of toxic chemicals that would lead to penalties and high restoration costs [3]. Hence, toxic emissions intensity in productions and operations is a critical concern to manufacturers [4]. It has drawn massive attention from both researchers and practitioners to investigate how the firm performance is impacted by environmental management e.g., [5,6], as well as the toxic and chemicals emissions, e.g., [7,8]. Previous studies have found a significant positive impact on stock returns from the environmental performance in the manufacturing industries, e.g., [3,9]. Furthermore, some recent studies, e.g., [10,11,12] have discussed that environment management develops a learning infrastructure in organizations to exploit existing knowledge for renewing and developing a green organizational capability, enabling firms to adapt more readily to the environment.

Specifically, many organizations increasingly develop knowledge-intensive processes for their competitiveness [13], and they tend to explore external sources of information to improve their performance [14,15]. Previous studies, e.g., [16,17,18] have demonstrated that absorptive capacity enables firms to improve innovation and financial performance based on the absorptive capacity is the ability of firms to recognize, gather, and absorb new external information as well as exploiting their existing internal information into new knowledge. In addition, from the operations management perspective, the manufacturing operation is responsible for transforming inputs into outputs in an efficient and effective manner [19]. Although slack resources and capacities have been suggested as a pool of necessary resources in an organization to achieve efficient and effective operation [20,21], previous studies on the relationship between the buffer inventory and firm performance offered mixed findings. Slack resources can be considered as excess inputs, unused capacity, and unnecessary expenditures [21,22], which could have been used to develop new products for entering a new market [22] and to react to environmental uncertainty and supply chain disruptions [23]. A firm’s buffer inventory resources can be used to fill the gap between supply and demand [19,24] and protect firms against operational glitches [23,25]. Recent studies, e.g., [24,26] suggested that buffer inventory is associated with improved performance in an unstable market.

The impacts of emissions reduction, absorptive capacity, and buffer inventory on firm performance have been largely discussed in previous research. Some studies have further examined the relationship between the absorptive capacity and environment management or the relationship between the buffer inventory and environmental management. From the resource-based view (RBV) of a firm, the absorptive capacity is a hardly imitable organizational learning capability that supports firms to acquire external information and exploit internal information over time to create a sustainable competitive advantage, e.g., [27,28]. Based on the RBV, organizational resources such as the buffer inventory can also provide the opportunity for firms to sustain their business in the competitive environment e.g., [29,30]. Yet, there is rather little understanding of any organizational mechanism that can link such firm-specific capabilities and resources to a sustainable competitive advantage. Therefore, the main question here is whether researchers and practitioners are missing the essential organizational mechanism for their competitiveness enhancement by solely investing in the absorptive capacity and buffer inventory. In particular, the resource orchestration perspective (ROP), which is an extension of the RBV, emphasizes that the possession of firm-specific capabilities and resources alone may not be enough for firms to gain a sustainable competitive advantage [31,32]. More importantly, firms need to develop a capability to orchestrate their resources, bundle them to form new capabilities, and use them to create competitive advantages [33,34]. The ROP suggests that an organizational process of coordinating and orchestrating firms’ strategic resources is a critical element to extract the values of the resource-based competitive advantage [34]. Through the organizational learning perspective, firms achieve low toxic emissions through specific systematic organizational routines. At the same time, they also develop systems that are favorable to acquire and share knowledge among employees for modifying their current practices to respond to the environment. Through the ROP, this study seeks to understand the toxic emissions produced in the operational routines as a critical organizational mechanism for orchestrating and deploying absorptive capabilities and buffer inventory resources to generate a sustainable competitive advantage, further enhancing firms’ market values. For researchers, it is important to explore the potential of existing firms’ capabilities and resources through any organizational mechanism for further business improvements. For practitioners, it is important to understand substantial requirements to extract values from their existing capabilities and resources to sustain and expand their business. In this study, we examine the impacts of the absorptive capacity and buffer inventory on the firm value and seek to understand the role of toxic emissions reduction in its interactions with absorptive capacity and buffer inventory for improving the market value of a firm. Using a sample of 391 manufacturing firms in the U.S. over the period of 2011 to 2018, we find that firms with low toxic emissions gain higher market value when they possess the absorptive capacity and the buffer inventory. The interaction among low toxic emissions, high absorptive capacity, and high buffer inventory can also lead to the highest firm value. 

The paper is structured as follows: Section 2 introduces the theoretical background and hypothesis development. Section 3 provides the research sample and methodology. Section 4 presents the test results. Section 5 discusses the contributions, theoretical and managerial implications. Section 6 concludes our research and discusses limitations and future research opportunities.

## 2. Theoretical Background and Hypothesis Development

### 2.1. Absorptive Capacity and Firm Value

Absorptive capacity is a firm’s learning capability to recognize, assimilate, transform, and exploit internal and external information, e.g., [16,18,35]. Previous studies have stated that absorptive capacity helps firms develop organizational capabilities such as the abilities to implement new operating practices to reduce production cycle time [35], to enhance product innovation [36], and to improve supply chain collaboration [37], thus leading to better performance. Based on the RBVof a firm, the absorptive capacity is a difficult-to-imitate organizational capability to create competitive advantages over time e.g., [27,28]. Firms with high absorptive capacity are more likely to achieve manufacturing flexibility benefits [18] and rapidly identify new opportunities [36,38]. Furthermore, firms with a stronger absorptive capacity usually have more interactions with their employees and suppliers as well as involvement in cross-functional activities [39,40], thus enriching data and information to improve their response to uncertainties. Specifically, absorptive capacity is viewed as a dynamic capability of RBV, e.g., [41,42,43] that is embedded in organizational routines and processes to integrate, develop, and reconfigure internal and external knowledge resources to develop sustainable competitive advantages [44,45]. In contrast, firms with low absorptive capacity are limited to identify, assimilate, and exploit their information to sustain their business. Overall, firms with high absorptive capacity are more capable of structuring and bundling their resources into competitive edges, resulting in a higher firm value.

**H1:**  *High absorptive capacity enhances firm value*.

### 2.2. Buffer inventory and Firm Value

Buffer inventory is considered to be firm’s resource buffers in response to demand variations [19,24] to protect firms against operational glitches [23,25]. Taking the RBV perspective, organizational resources hold the potential of a sustainable competitive advantage to support business growth, e.g., [29,30]. Buffer inventory allows a firm to tactically utilize assets to manage mismatches between supply and demand, which gives a firm a competitive advantage to survive in a fast-changing market [24]. Previous studies have found that slack resources enhance firm performance, e.g., [46,47]. Firms with a greater buffer inventory are likely to have more flexibility to react to the market, reduce supply chain disruptions, and offer more product variety to satisfy customers, thus improving their market positions and leading to a higher firm value [47,48].

**H2:**  *High buffer inventory enhances firm value*.

### 2.3. Toxic Emissions

Manufacturing firms that release toxic chemicals face increased exposure to regulatory requirements, compliance costs, and risks to damage their corporate image [4]. This stimulates manufacturers to reconfigure their processes and systems to minimize toxic emissions, to save energy and natural resources, as well as to protect communities, employees, and consumers [4,49]. Firms with low toxic emissions are more capable of understanding how operations interact with the environment and addressing various environmental impacts associated with daily processes [50,51]. In particular, they develop an organizational structure with systematic routines for using and sharing knowledge across functional areas, thus enhancing employees’ awareness of the importance of reducing toxic emissions and the environmental impacts of organizational products and services [52,53]. This helps to reduce employees’ resistance to adopting the new green strategies [54]. A firm with the organizational infrastructure to encourage employee engagement is more open and willing to learn new knowledge, take up new challenges, and adjust to the environment [55,56]. Accordingly, we argue that firms with low toxic emissions are more likely to bundle the information that is acquired and exploited by their absorptive capacity into daily operations to facilitate organizational learning, thus leveraging knowledge to create benefits to firms.

In addition, firms achieving low toxic emissions through their systematic operational routines develop a shared understanding, which might support firms in using buffer inventory for operational efficiency, enhancing agility and leading to a higher market value. From the ROP of the RBV, it is more important that firms possess the capability to orchestrate their resources, bundle them to develop new capabilities, and use them to achieve competitive advantages [33,34]. The possession of strategic resources alone may not be enough for firms to sustain business performance in the competitive market [31,32]. The ROP suggests that the organizational process of structuring resources such as the buffer inventory is a critical element for deriving the values of the resource-based competitive advantage [34]. To reduce toxic chemicals released in productions by establishing a systematic operational system is likely to put firms in a better position to deploy buffer inventory resources, thus orchestrating and integrating operational capabilities for reducing toxic emissions in the managerial resource allocation decision-making process. Therefore, firms with low toxic emissions might be more capable of deploying the buffer inventory strategically to achieve a significant competitive advantage, which leads to a higher firm value.

**H3a:**  *The positive impact of the absorptive capacity on the firm value is strengthened through low toxic emissions*.

**H3b:**  *The positive impact of the buffer inventory on the firm value is strengthened through low toxic emissions*.

### 2.4. Absorptive Capacity, Buffer inventory, and Toxic Emissions

Firms with low toxic emissions usually make continuous efforts to improve their environmental performance [6]. They establish organizational routines to streamline the information collection process and encourage open communication to acquire more internal and external data. They emphasize using resources efficiently and reducing waste. Absorptive capacity relies on structured organizational routines [57] that facilitate firms to exploit existing knowledge embedded in their systems into new knowledge, and then integrate and reconfigure internal and external knowledge resources. Firms pursuing low toxic emissions together with a high absorptive capacity are more likely to use the information for continuous improvements, thus enabling them to have updated and insightful knowledge to address operational discrepancies and environmental uncertainties. A high absorptive capacity and a low toxic chemical release might not be enough for a firm to yield its market value to the greatest extent. In fact, the firm also needs to prepare resource buffers to stabilize any fluctuations in its product demands, production capacities, and lead times to deliver good customer service. Overall, organizational toxic emissions, absorptive capacity, and buffer inventory are interrelated to connect firms with their internal and external environments, enabling them to be in a better position to align resources and to renew capabilities over time to be more competitive in a dynamic market.

**H4:**  *The interaction of high absorptive capacity, high buffer inventory, and low toxic emissions leads to the highest firm value*.

## 3. Methodology

### 3.1. Sample and Data Collection

We sampled U.S. listed manufacturing firms (SIC 2000–3999) during the period of 2011–2018. We collected financial data from the Compustat database which is provided by Wharton Research Data Services in Philadelphia and toxic emissions data from the U.S. Environmental Protection Agency (EPA)’s Toxic Release Inventory (TRI) database. The TRI database has been widely used to measure toxic emissions, e.g., [58,59,60,61]. It covers over 700 chemicals that harm the natural environment or human health. The EPA classifies the chemicals into several categories, such as cyanide compounds, dioxin and dioxin-like compounds, lead compounds, mercury compounds, nitrate compounds, and sodium nitrite, which are included in our study. Every U.S. facility with ten or more full-time employees has to report releases and transfers of each chemical above a specified threshold on an annual basis. We obtained 391 sample firms that have sufficient data to measure their absorptive capacity, buffer inventory, firm value in terms of Tobin’s Q, and toxic emissions. Table 1 presents the distribution of the sample firms based on their 2-digit SIC codes. Most of the sample firms are in industries related to industrial machinery and equipment, electronic and other electric equipment, as well as chemical and allied products.

### 3.2. Measurements

In this study, we argue that the absorptive capacity and buffer inventory are important organizational capability and resource to create a firm’s sustainable competitive advantage through the RBV of a firm and dynamic capability perspectives. More importantly, we argue that the organizational initiative on reducing toxic emissions can further derive the values of the absorptive capacity and buffer inventory to improve the firm value from the organizational learning perspective. Accordingly, first, we examine the impacts of two independent variables, absorptive capacity and buffer inventory, on the dependent variable of firm value. Taking the perspective on the RBV, absorptive capacity is a difficult-to-imitate firm’s capability to develop competitive advantages over time, e.g., [27,28]. In addition, absorptive capacity is viewed as a dynamic capability, e.g., [59,60], which extends the RBV [43] to emphasize firms’ reconfiguration of their internal and external resources to create sustainable competitive advantages in the dynamic environment, e.g., [53,54]. Similarly, the RBV suggests that organizational resources hold the potential of a sustainable competitive advantage to support business growth, e.g., [29,30]. Inventory buffer resources enable firms to meet up with demand volatility to maintain their competitive advantages [24,62]. Therefore, we argue that the absorptive capacity and inventory buffer are the firm-specific capability and resource that might generate value to firms in terms of Tobin’s Q. Second, we explore the role of toxic emissions in the impacts of the absorptive capacity and buffer inventory on the firm value. Firms implement environmental initiatives such as pollution prevention, waste reduction, and materials recycling to enhance their performance [63]. Firms with low toxic emissions through systematic routines can develop an organizational learning infrastructure to support open communication and share knowledge [52,53]. As such, we investigate whether the low toxic emissions in the learning organization can better utilize the knowledge through the absorptive capacity, thus further enhancing the firm value. Furthermore, firms with low toxic emissions encourage good communications among employees, facilitating them to gain a better understanding of current best practices and new knowledge [55,56]. Thus, we need to study whether the low toxic emissions enable firms to better allocate and use their buffer inventory to create a sustainable competitive advantage. The measurements of each variable are introduced in the following sub-sections.

#### 3.2.1. Firm Value

Following prior studies, e.g., [64,65,66,67], we measured a firm’s value based on Tobin’s Q. Since Tobin’s Q takes all the available information of a company to investors into account, it is a market-based and forward-looking measure of a firm’s value [65,68]. This measure fits this research well because firms investing in the reduction of toxic chemicals in their productions and operations and the developments of absorptive capacity and buffer resources can influence their future market value. We employed the widely used approach by Chung and Pruitt [69] to measure Tobin’s Q of firm *i* in year *t* as expressed in the following Equation (1):

(1) $$\begin{array}{ll} Tobin^{\prime }s\;Q_{it}= & (Common\;Shares\;Outstanding_{it}\times Share\;Price_{it}\\ & \;+Liquidation\;Value\;of\;Preferred\;Stock_{it}\\ & \;+Long-term\;Debt_{it}+Current\;Liabilities_{it}\\ & \;-Current\;Assets_{it})/Total\;Assets_{it} \end{array}$$

#### 3.2.2. Absorptive Capacity

Absorptive capacity is the ability of a firm to recognize, assimilate, transform, and exploit knowledge from its dynamic environment [16,70]. We used the R&D intensity, which is the ratio of R&D expenditures to sales, to measure a firm’s absorptive capacity [16,71].

#### 3.2.3. Buffer inventory

The available buffer inventory resources support a firm’s operational activities, allowing a firm to effectively manage demand variations [24] to support firms to better match supply and demand [19,24]. We measured a firm’s buffer inventory based on the number of inventory days [19,23].

#### 3.2.4. Toxic Emissions

Toxic emissions are measured as a reversed toxic emission intensity. In other words, a firm has low toxic emissions if its reversed toxic emission intensity is greater. The toxic emission intensity is the natural logarithm of a ratio of total toxic emission amounts to sales plus one [61,72]. We obtained the total toxic emission amounts of firms from the TRI database, e.g., [58,59,60,61].

#### 3.2.5. Control Variables

In this research, we consider several control variables, including firm age, firm size, sales growth, and labor intensity. We measure the firm age as the natural logarithm of the number of years from the incorporation date [73,74]. Older firms usually have more experience, abilities, and skills to establish better technology, effective supply chain, and good customer relationships, and they can also derive more benefits from their accumulated knowledge, thus might achieve a better performance to improve the firm value [75]. We take the firm size as the natural logarithm of total assets [76,77]. Previous research has shown that the firm size is negatively related to Tobin’s Q [78,79]. We measure the sales growth of firms as the rate of growth in sales revenue [73,80]. Firms with a higher sales growth are usually perceived as having higher sales potency and better business prospects to enhance the firm value [81,82]. Finally, we measure the labor intensity as a ratio of employee number to total assets [68,83]. A greater labor-intensive firm tends to rely more on the skills and competency of the workforce, so it runs a higher risk of generating more defects in operations [84], thus might deteriorate the firm reputation and its future value.

Table 2 summarizes all the variables along with their sources used in this research.

### 3.3. Statistical Analyses

We use a fixed-effect regression model to examine the impacts of the absorptive capacity and buffer inventory on the firm value and the moderating effect of toxic emission intensity based on the significant Hausman test results (*p* < 0.01). In this research, we include firm-level control variables such as firm age, firm size, sales growth, and labor intensity, that may affect firm value. Yet, some potential confounding factors related to firm characteristics such as leadership style may exist and influence a firm to take initiatives to improve its absorptive capacity, buffer inventory, and environmental performance, such as the amount of toxic chemicals a firm releases to the environment, affecting its market value. Using the fixed-effect regression model, we can address this endogeneity issue by incorporating a firm-level fixed effect estimation to remove unobservable firm-specific factors [85,86]. In addition, some unobservable time-specific factors such as economic environment and industry trends may affect the firm value over time. Thus, the fixed-effect regression model includes a year-level fixed-effect estimation to remove unobservable time-specific impacts [85,86]. As the fixed-effect regression model also can remove any industry-level effects to avoid heterogeneity across industries [87], our regression model does not control for industry-level factors in order to be consistent, as in previous studies.

Furthermore, we use a one-year lag between the dependent variable, firm value (measured in year *t* + 1) and the independent variables such as the absorptive capacity and buffer inventory (measured in year *t*) to ensure the direction of causality.

Our fixed-effect regression model is as expressed in the following Equation (2):

(2) $$\begin{array}{ll} Tobin^{\prime }s\;Q_{i,t+1}= & \beta_{0}+\beta_{1}Firm\;Age_{it}+\beta_{2}Firm\;Size_{it}+\beta_{3}Sales\;Growth_{it}\\ & +\beta_{4}Labor\;Intensity_{it}+\beta_{5}Absorptive\;Capacity_{it}\\ & +\beta_{6}Buffer\;Inventory_{it}+\beta_{7}Toxic\;Emissions_{it}\\ & +\beta_{8}\left(Absorptive\;Capacity_{it}\times Toxic\;Emissions_{it}\right)\\ & +\beta_{9}\left(Buffer\;Inventory_{it}\times Toxic\;Emissions_{it}\right)\\ & +\beta_{10}\left(Absorptive\;Capacity_{it}\times Buffer\;Inventory_{it}\right)\\ & +\beta_{11}(Absorptive\;Capacity_{it}\times Buffer\;Inventory_{it}\\ & \times Toxic\;Emissions_{it})+\alpha_{i}+\delta_{t}+\varepsilon_{it} \end{array}$$

where *i* refers to the *i*th sample firm and *t* refers to the year *t*; $\alpha_{i}$, $\delta_{t}$, and $\varepsilon_{it}$ are the firm-level fixed effects, year-level fixed effects, and the error term, respectively.

In our analyses, $\beta_{5}$ estimates the impact of the absorptive capacity on the firm value (H1), $\beta_{6}$ estimates the impact of the buffer inventory on the firm value (H2), $\beta_{8}$ and $\beta_{9}$ estimate the moderating role of toxic emissions in the relationship between absorptive capacity and firm value (H3a) and the relationship between buffer inventory and firm value (H3b), respectively, and $\beta_{11}$ estimates the interacting effect of the absorptive capacity, buffer inventory, and toxic emissions on firm value (H4).

## 4. Results

Table 3 shows the descriptive statistics and correlations of the variables in this research. Table 4 presents the fixed-effect regression test results. Model 1 is the base model including all control variables, firm-level fixed effect, and year-level fixed effect. Model 2 adds the two main effects of the independent variables of absorptive capacity and buffer inventory. Model 3 adds the moderating effects of toxic emissions. Model 4 adds the three-way effect of absorptive capacity, buffer inventory, and toxic emissions. All four models are statistically significant (*p* < 0.01) based on the F-tests with their R-squares ranging from 0.010 to 0.163. The low R-squared values are due to several insignificant control variables in the base model, while the changes in the R-squared values of 0.113 for Model 2, 0.14 for Model 3, and 0.153 for Model 4 compared to the base model indicate that the explanatory powers increase by adding absorptive capacity, buffer inventory, toxic emissions, and the interaction terms. In addition, all four models are statistically significant (*p* < 0.01) based on the Hausman test, indicating that the fixed effects model is more appropriate than the random effect model to use. The four models are estimated based on 2808 observations of 391 samples, suggesting that there are about seven observations for each of the 391 sample firms (unbalanced panel).

The control variable, labor intensity, remains significantly negative (*p* < 0.01) to the firm value across the four models, suggesting that higher labor-intensive firms have a lower firm value. Another control variable, firm age, is significantly positive (*p* < 0.05 in Models 2 and 4; *p* < 0.01 in Model 3) to the firm value across Models 2 to 4, indicating that older firms have a better firm value.

The effect of the absorptive capacity is significantly positive (*p* < 0.01) across Models 2 to 4, suggesting that firms with a stronger absorptive capacity have a better firm value. Thus, H1 is supported. Although the effect of the buffer inventory on the firm value is significantly negative (*p* < 0.05) as shown in Model 3, the effect of the buffer inventory is insignificantly negative (*p* > 0.1) as shown in Models 2 and 4. Thus, H2 is not supported.

**Figure 1:**
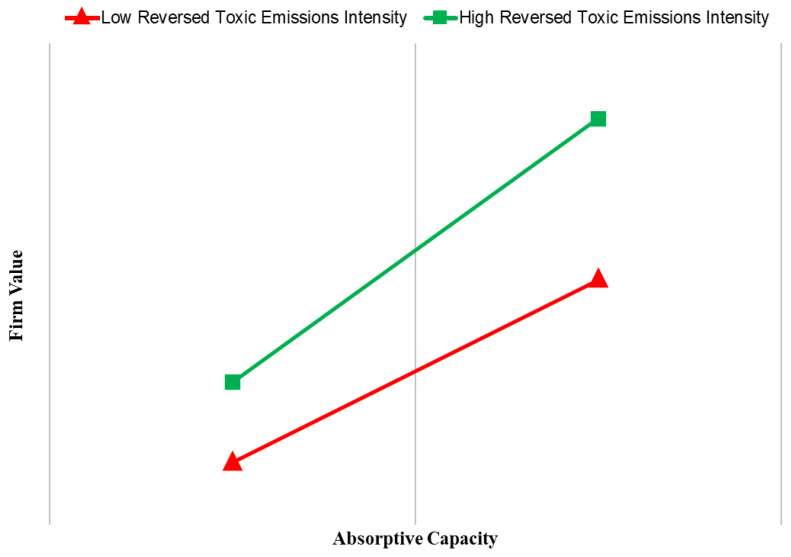
Moderating effect of toxic emissions (i.e., the reversed toxic emissions intensity) on the relationship between absorptive capacity and firm value.

The reversed toxic emissions intensity provides a significantly positive impact on the firm value (*p* < 0.01) in Models 3 and 4. Models 3 and 4 also indicate that the interaction between the absorptive capacity and the reversed toxic emissions is positive and significant (*p* < 0.1 in Model 3 and *p* < 0.05 in Model 4). Following Aiken et al. [88], we plot the relationship between the absorptive capacity and firm value at high and low values (±1 standard deviation) of the reversed toxic emissions intensity in Figure 1. The plot shows that the slope of the relationship between absorptive capacity and firm value is more positive (*p* < 0.01) for firms with a high effort to reduce toxic emissions than firms with a low effort to reduce toxic emissions. In other words, higher effort made in the reduction of toxic emissions further enhances the positive relationship between the absorptive capacity and firm value. Thus, H3a is supported.

**Figure 2:**
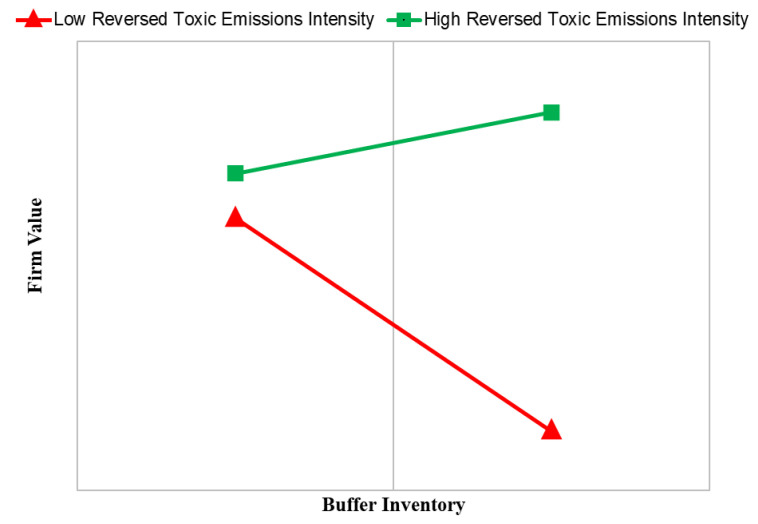
Moderating effect of toxic emissions (i.e., the reversed toxic emissions intensity) on the relationship between buffer inventory and firm value.

The interaction between the buffer inventory and the reversed toxic emissions intensity is positive and significant (*p* < 0.01) in Models 3 and 4. Figure 2 illustrates the relationship between the buffer inventory and firm value at high and low values (±1 standard deviation) of the reversed toxic emissions intensity. The plot shows that the slope of the relationship between the buffer inventory and firm value is positive (*p* < 0.05) for firms with a high effort to reduce toxic emissions while the slope of the relationship between buffer inventory and firm value is negative (*p* < 0.01) for firms with a low effort to reduce toxic emissions. In other words, a higher effort made to reduce toxic emissions reverses the negative impact of the buffer inventory on the firm value. Thus, H3b is supported.

**Figure 3:**
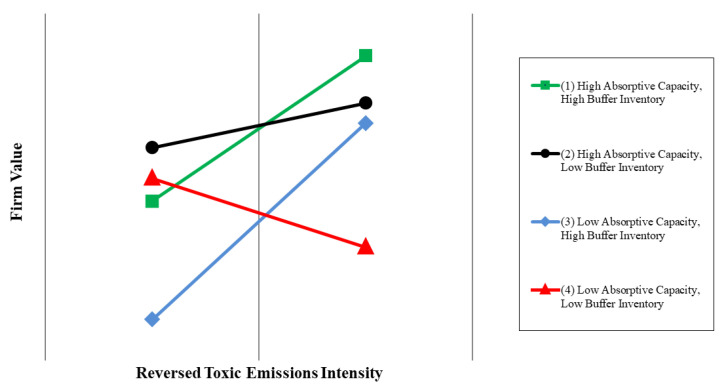
Three-way interaction of toxic emissions (i.e., the reversed toxic emissions intensity), absorptive capacity, and buffer inventory.

Model 4 presents the result that the three-way interaction among absorptive capacity, buffer inventory, and toxic emissions on the firm value are negative and significant (*p* < 0.01). The three-way interaction is complex, because the relationship between toxic emissions and the firm value is contingent not only on the absorptive capacity but also on the buffer inventory. Previous studies, e.g., [88,89,90] have suggested that plotting the interaction is an efficient way to interpret the three-way interaction. Figure 3 illustrates the relationship between toxic emissions in terms of the reversed toxic emissions intensity and the firm value at high and low values (±1 standard deviation) of absorptive capacity and buffer inventory. When firms have a high absorptive capacity, the relationship between the reversed toxic emissions intensity and firm value is significant and more positive if firms also have a high buffer inventory (i.e., line 1; *p* < 0.01). In contrast, if firms have a low absorptive capacity, the relationship between the reversed toxic emissions intensity and firm value is significant and negative if firms also have a low buffer inventory (i.e., line 4; *p* < 0.01). Furthermore, as indicated on line 1, the firms that have high values of all absorptive capacity, buffer inventory, and the reversed toxic emissions intensity gain the highest firm value. In contrast, as indicated on line 4, the firms that have low values of all absorptive capacity, buffer inventory, and the reversed toxic emissions intensity obtain the lower firm value. These results support H4, which states that firms investing greater effort in the reduction of toxic emissions gain a higher firm value when their absorptive capacity and buffer inventory are both at a high level.

## 5. Discussion

We empirically examined the impacts of absorptive capacity and buffer inventory on the market value of firms with low toxic emissions. The results of our fixed-effect regression analysis based on a sample of 391 U.S. manufacturing firms between 2011 and 2018 indicates that the absorptive capacity leads to a higher firm value, especially for firms with low toxic emissions. Conversely, the impact of the buffer inventory on the firm value is insignificant. Such an impact will become significant and leads to a higher market value when firms achieve low toxic emissions. Furthermore, our results show that the highest market value can be achieved if a firm has low toxic emissions, a high absorptive capacity, and high buffer inventory. The main contribution of our study is to provide empirical evidence that the low-level release of toxic chemicals is an important contextual factor for firms to realize the benefits of absorptive capacity and buffer inventory. The understanding of this contextual factor is even enhanced as firms are more likely to gain better performance from deploying absorptive capacity and buffer inventory. We discuss the theoretical and practical implications below.

### 5.1. Theoretical Implication

In this study, we have considered the reduction of toxic emissions from the organizational learning perspective to guide an organization through its efforts to understand the process and then orchestrate the available resources to the process. Based on the RBV, firm’s capabilities such as its absorptive capacity and its buffer inventory resources are valuable to lead to firm competitiveness. Firms with low toxic emissions might be able to bundle the acquired information into daily operational routines and to develop a learning environment for encouraging employees to share and use the information. So, firms use the updated knowledge to improve their existing processes to create competitive advantages. Academics have long discussed the importance of information and knowledge for improving organizational routines and competencies [13]. However, the role of toxic emissions reduction in the impacts of the absorptive capacity and buffer inventory on firm value has been studied sparsely. Previous studies, e.g., [16,17,18] have stated that absorptive capacity leads to better innovation and financial performance. On the other hand, the impact of slack resources on firm performance is rather controversial, e.g., [20,21,26]. Our study provides substantial empirical evidence that the impacts of low toxic emissions with the absorptive capacity and buffer inventory are both significantly positive on the firm value. There has always been a lack of understanding about the specific organizational context in which the benefits from absorptive capacity and buffer inventory can be realized. We contribute to the understanding from the organizational learning perspective by exploring the role of toxic emissions reduction in the utilization of information, which is acquired by the absorptive capacity and the alignment of buffer inventory resources, to maximize the firm’s returns. In particular, the ROP of the RBV pinpoints the importance of an organizational process of coordinating and orchestrating firms’ resources for extracting more benefits of the resource-based competitive advantage. This study shows that the reduction of toxic emissions through operational processes is a critical factor for making the understanding of ROP more salient.

### 5.2. Practical Implication

We have pointed out that firms actually can increase their market value by keeping their toxic emissions at a low level and put significant efforts into deploying absorptive capacity and buffer inventory at the same time. Firms committed to reducing toxic emissions have well-established systems, stable operational routines, and efficient information flow. All of these lead to a stronger organizational learning capability. Such systematic organizational practices also enable firms to orchestrate their existing capabilities and resources, and integrate and use them to modify the operational activities to deal with new challenges and lead to a sustainable competitive advantage. When firms achieve low toxic emissions together with a high absorptive capacity, they are equipped with a stronger learning capability to acquire, share, and use internal and external information continuously. They can therefore evaluate and renew their operating contexts quickly to respond to the changes in the environment. And they might also be capable of developing more robust operational routines to support managers in identifying and using buffer inventory resources efficiently and effectively. These routines reduce uncertainties in the internal and external environments, thus realizing potential benefits from using a buffer inventory. More importantly, these firms manage to excel in their management of the absorptive capacity and buffer inventory at the same time. Through the orchestration process, the potential values of firm-specific capabilities and resources can be actualized and enhanced. As a result, the established organizational learning infrastructure through the reduction of emissions facilitates firms to efficiently acquire, transform, and integrate internal and external information into new knowledge. The stable operational routines also support them in aligning buffer inventory resources and in modifying existing organizational capabilities over time for the further enhancement of the firm competitiveness. Firms do not only generate strategic resources and capacities to create competitive advantages, but also need to develop their own ability to coordinate their resources and capacities to strengthen existing practices to advantage their competitiveness.

## 6. Conclusions

This research contributes to the understanding of the impacts of absorptive capacity and buffer inventory on firm value as well as on integrating toxic emissions intensity. We particularly enrich the research on green organizational strategies in the manufacturing industries. By taking the organizational learning perspective, we suggest that the manufacturing firms with low toxic emissions develop systematic operational routines for sharing and using information, thus improving goal alignment and learning capabilities for firms to adapt more readily to the changing environment. Accordingly, firms with low toxic emissions have established a learning infrastructure that facilitates firms to bundle the new knowledge that is acquired and exploited by their absorptive capacity into daily routines. They also encourage their employees to deploy the new knowledge to modify current practices and use a buffer inventory in an effective and efficient manner. As a result, low toxic emissions can provide firms with a more favorable condition to leverage the values of absorptive capacity and buffer inventory. The firms are also in a better position to align resources and renew capabilities over time to be more competitive in the dynamic environment. Previous studies have examined the impacts of emissions reduction, absorptive capacity, and buffer inventory resources on the firm performance, while the research on slack resources led mostly to mixed findings. Few studies have investigated the role of toxic emissions intensity in the impacts of absorptive capacity and buffer inventory on firm value from the organizational learning perspective. Our research demonstrates that firms with low toxic emissions gain higher market value when they possess the absorptive capacity and the buffer inventory. In addition, the interaction among low toxic emissions, high absorptive capacity, and high buffer inventory significantly leads to the highest firm value.

There are some limitations in this study which could become some possible directions for future research. This study focused on toxic emission and the emission data were collected from the U.S. EPA’s TRI database. Since only data on toxic emissions are provided by the database, the scope of emission is limited to toxic chemicals. Further research can be conducted to examine the difference between the effects of toxic and non-toxic emissions on the impacts of the absorptive capacity and buffer inventory to the firm value. We used the R&D intensity as a proxy for absorptive capacity. Although the R&D investment of firms is commonly used as an indicator for an organization’s capability to explore and exploit information, the effectiveness in each process to recognize, assimilate, transform, and exploit information of their absorptive capacity may differ from firm to firm. Future research can examine and compare the impact of the potential as well as realized absorptive capacity on the firm value [91,92]. The potential absorptive capacity is related to the acquisition and the assimilation capabilities, while the realized absorptive capacity is related to capabilities of transformation and exploitation. Our sample is limited to the S&P 500 Index in the U.S., so researchers can further examine the role of toxic emissions intensity on the absorptive capacity and buffer inventory in different contexts for small and medium-sized enterprises. Furthermore, this research relies on secondary data to investigate our research questions. Finally, future studies can focus on exploring other factors such as supply chain capability and technology competence [93,94] that may affect firms’ learning capabilities and enable them to be more competitive.

## Figures and Tables

**Table 1:** Distribution of sample firms across industries.

2-Digit SIC Code	Industry	Frequency	Percentage
35	Industrial Machinery and Equipment	61	15.60
36	Electronic and Other Electric Equipment	58	14.83
28	Chemical and Allied Products	55	14.07
37	Transportation Equipment	43	11.00
38	Instruments and Related Products	35	8.95
34	Fabricated Metal Products	27	6.91
20	Food and Kindred Products	21	5.37
33	Primary Metal Industries	16	4.09
26	Paper and Allied Products	14	3.58
29	Petroleum and Coal Products	13	3.32
30	Rubber and Miscellaneous Plastics Products	12	3.07
25	Furniture and Fixtures	10	2.56
32	Stone, Clay, and Glass Products	9	2.30
Other SIC Codes	Other Industries	17	4.35
Total		391	100

**Table 2:** Variable descriptions.

Variables	Measurements	Data Sources	References
Firm Value	Use Tobin’s Q as a proxy for firm value, Tobin’s Q = $\frac{\begin{array}{c} Common\;shares\;outstanding\times Share\;price+Liquidation\;value\;of\;preferred\;stock\;\\ +Long-term\;debt+Current\;liabilities-Current\;asset \end{array}}{Total\;asset}$	Compustat	[66,69]
Absorptive Capacity	Measured as R&D intensity, R&D intensity = $\frac{R\&D\;expenditures}{Sales}$	Compustat	[16,71]
Toxic Emissions	Use reversed toxic emission intensity as a proxy for toxic emissions, Toxic emission intensity = *LN*($\frac{Total\;toxic\;emission\;amounts}{Sales}+1$)	The U.S. EPA’s Toxic Release Inventory (TRI)	[59,61]
Buffer Inventory	The number of inventory days	Compustat	[19,23]
Firm Age	The natural logarithm of the number of years since incorporation date	Compustat	[73,74]
Firm Size	The natural logarithm of total assets	Compustat	[76,77]
Sales Growth	The rate of growth in sales revenue	Compustat	[73,80]
Labor Intensity	The employee number divided by total assets	Compustat	[68,83]

**Table 3:** Descriptive statistics and correlations.

Variables	1.	2.	3.	4.	5.	6.	7.	8.
Firm Value	1							
2. Absorptive Capacity	0.269 ***	1						
3. Buffer Inventory	0.051 ***	0.162 ***	1					
4. Toxic Emissions (i.e., the reversed toxic emissions intensity)	0.154 ***	0.196 ***	0.185 ***	1				
5. Firm Age	0.037 **	−0.079 ***	0.040 **	0.022	1			
6. Firm Size	0.076 ***	0.054 ***	0.033 *	−0.003	0.139 ***	1		
7. Sales Growth	0.037 *	0.006	−0.030	−0.014	−0.043 **	−0.018	1	
8. Labor Intensity	−0.151 ***	−0.031 *	0.005	0.219 ***	0.025	−0.328 ***	−0.041 *	1
Mean	1.298	0.037	87.328	−2.948	4.956	7.758	0.082	0.004
Standard deviation	1.036	0.089	60.824	2.297	0.882	1.736	0.686	0.002
Minimum	−0.453	0.000	7.021	−9.115	1.000	2.139	−0.631	0.000
Maximum	18.858	1.831	580.160	0.000	6.361	12.764	34.127	0.018

Notes: * *p* < 0.1, ** *p* < 0.05, *** *p* < 0.01 (two-tailed tests).

**Table 4:** Fixed-effect regression test results.

Independent Variables	Dependent Variable: Firm Value			
	Model 1 (Control)	Model 2 (H1 and H2)	Model 3 (H3a and H3b)	Model 4 (H4)
Intercept	0.292 *** (0.055)	0.233 *** (0.052)	0.249 *** (0.051)	0.243 *** (0.051)
Firm Age	0.016 (0.023)	0.057 ** (0.023)	0.059 *** (0.022)	0.052 ** (0.022)
Firm Size	−0.001 (0.013)	−0.011 (0.012)	−0.022 * (0.012)	−0.012 (0.012)
Sales Growth	−0.003 (0.013)	0.022 (0.026)	0.022 (0.026)	0.022 (0.026)
Labor Intensity	−46.188 *** (11.582)	−61.973 *** (11.197)	−70.385 *** (11.077)	−71.562 *** (11.012)
Absorptive Capacity		3.532 *** (0.211)	2.793 *** (0.334)	2.279 *** (0.347)
Buffer Inventory		−0.000 (0.000)	−0.001 ** (0.000)	−0.000 (0.000)
Toxic Emissions (i.e., the reversed toxic emissions intensity)			0.064 *** (0.010)	0.065 *** (0.010)
Absorptive Capacity × Toxic Emissions (i.e., the reversed toxic emissions intensity)			0.247 * (0.134)	0.292 ** (0.134)
Buffer inventory × Toxic Emissions (i.e., the reversed toxic emissions intensity)			0.001 *** (0.000)	0.002 *** (0.000)
Absorptive Capacity × Buffer Inventory				0.000 (0.006)
Absorptive Capacity × Buffer Inventory × Toxic Emissions (i.e., the reversed toxic emissions intensity)				−0.009 *** (0.002)
Firm-Level Fixed Effects	Included	Included	Included	Included
Year-Level Fixed Effects	Included	Included	Included	Included
*F*-statistic	2.430 ***	27.422 ***	27.767 ***	26.853 ***
*R*-squared	0.010	0.123	0.150	0.163
Adjusted *R*-squared	0.006	0.118	0.145	0.157
Hausman Test	*p* < 0.01	*p* < 0.01	*p* < 0.01	*p* < 0.01

Note: * *p* < 0.1, ** *p* < 0.05, *** *p* < 0.01 (two-tailed tests), standard errors are in parentheses, one-year lag between the dependent variable and all independent variables, *n* = 391, number of observations = 2808.

## Data Availability

Not applicable.
